# Cytoskeletal Components of an Invasion Machine—The Apical Complex of Toxoplasma gondii

**DOI:** 10.1371/journal.ppat.0020013

**Published:** 2006-02-24

**Authors:** Ke Hu, Jeff Johnson, Laurence Florens, Martin Fraunholz, Sapna Suravajjala, Camille DiLullo, John Yates, David S Roos, John M Murray

**Affiliations:** 1 Department of Cell Biology, Scripps Research Institute, La Jolla, California, United States of America; 2 The Stowers Institute for Medical Research, Kansas City, Missouri, United States of America; 3 Institute of Microbiology, E.-M.-Arndt University, Greifswald, Germany; 4 Department of Biology, University of Pennsylvania, Philadelphia, Pennsylvania, United States of America; 5 Department of Anatomy, Philadelphia College of Osteopathic Medicine, Philadelphia, Pennsylvania, United States of America; 6 Department of Cell & Developmental Biology, University of Pennsylvania, Philadelphia, Pennsylvania, United States of America; Stanford University, United States of America

## Abstract

The apical complex of Toxoplasma gondii is widely believed to serve essential functions in both invasion of its host cells (including human cells), and in replication of the parasite. The understanding of apical complex function, the basis for its novel structure, and the mechanism for its motility are greatly impeded by lack of knowledge of its molecular composition. We have partially purified the conoid/apical complex, identified ~200 proteins that represent 70% of its cytoskeletal protein components, characterized seven novel proteins, and determined the sequence of recruitment of five of these proteins into the cytoskeleton during cell division. Our results provide new markers for the different subcompartments within the apical complex, and revealed previously unknown cellular compartments, which facilitate our understanding of how the invasion machinery is built. Surprisingly, the extreme apical and extreme basal structures of this highly polarized cell originate in the same location and at the same time very early during parasite replication.

## Synopsis

*Toxoplasma gondii,* the leading cause of human congenital neurological defects, is also closely related to the malaria parasite *Plasmodium falciparum.* To survive and multiply, these parasites must invade a host cell. Once inside the cell, the parasites quickly outgrow their host and then break out, destroying the host cell in the process. The damage they cause is almost entirely due to uncontrolled cycles of host-cell invasion, parasite multiplication, and host-cell destruction. Both the invasion of host cells and parasite multiplication seem to rely on a remarkable set of structures known as the apical complex. The authors thought that a better understanding of how the apical complex does its job might lead to new ideas for combating diseases caused by this family of parasites.

Unfortunately, the molecular components of the apical complex were unknown, which severely hampered study of its function. Hu et al. developed a procedure for identifying most of the proteins used to build the apical complex, and report the results in this paper. The work provides the basis for future studies of how these proteins work, and thus ultimately a basis for choosing targets against which more effective drugs could be designed.

## Introduction

Toxoplasma gondii is one of ~5,000 species of obligate intracellular protozoan parasites in the phylum *Apicomplexa,* many members of which are human or animal pathogens [1]. T. gondii is the most common cause of congenital neurological defects in humans, and a devastating opportunistic infection in immunocompromised patients. Other prominent members of the phylum include *Plasmodium falciparum,* the most lethal cause of malaria; *Eimeria sp.,* a poultry and cattle pathogen; *Cryptosporidia,* an opportunistic human and animal pathogen; and *Babesia* and *Theileria,* cattle parasites*.* Much of the pathogenesis associated with these parasitic diseases is due to tissue damage caused by uncontrolled cycles of host-cell invasion, parasite proliferation, host-cell lysis, and reinvasion. Understanding parasite invasion and replication is therefore essential for understanding parasitic disease.

**Figure 1:**
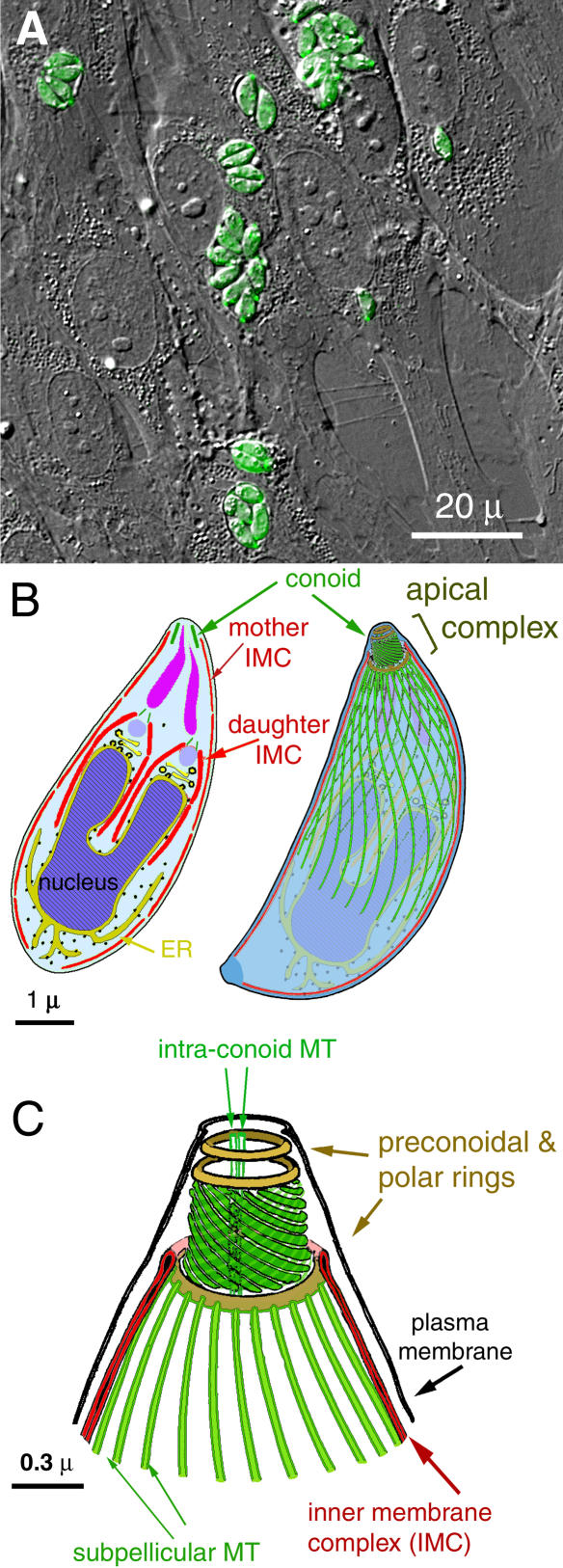
Image and Drawing of T. gondii (A) Combined DIC and epifluorescence image of human fibroblasts infected with transgenic T. gondii expressing GFP-tubulin (green). Parasitophorous vacuoles containing 1, 2, 4, or 8 parasites are seen. (B) Drawings of T. gondii (modified from [3] and [68]). Left: a longitudinal section of a dividing cell. Lobes of the dividing nucleus bordered by ER, Golgi (yellow), and developing rhoptry (mauve) are surrounded by the developing daughters' scaffolds (red). Maternal and daughter conoids are shown in green, secretory organelles (rhoptries) in purple. T. gondii has three membranes: a plasma membrane (black) and two additional layers (IMC, red) formed from a patchwork of flattened vesicles. Right: semitransparent view showing subpellicular MT (green). (C) Enlarged view of the apical complex cytoskeleton, showing the conoid (green), preconoidal, and polar rings (brown), and two intraconoid MT (green). The conoid is formed of 14 fibers of tubulin (not MT), 430 nm long, arranged in a left-handed spiral [10]. Cytoskeletal elements, including the subpellicular MT (green) and a 2-dimensional lattice of intermediate filament-like proteins (not shown), are closely associated with the cytoplasmic face of the IMC.

Most Apicomplexan parasites share a set of cytoskeletal structures essential for parasite survival and pathogenesis [2]. Central to both invasion and proliferation is a group of structures at one end of the adult parasite, the apical complex, from which the phylum derives its name. The apical complex, as shown for T. gondii in Figure 1, is built around the conoid, an assembly of spirally arranged fibers that is actively motile during invasion. Other apical complex structures closely associated with the conoid are 1) the preconoidal rings, at the distal tip of the conoid, from which the conoid fibers originate; 2) the polar ring, from which the parasite's 22 subpellicular microtubules (MT) originate; and 3) two short intraconoid MT, which may be used as tracks for transport of secretory vesicles essential for invasion [3,4]. The extension and retraction of the conoid associated with host cell invasion [5] suggests a mechanical role for the apical complex during attachment or penetration. In addition, regulated secretion from this region supplies factors essential for attachment, invasion, and subsequent formation of the parasitophorous vacuole in which the parasite encloses itself [6]. During parasite replication, the first sign of daughter formation is the de novo construction of daughter conoids and apical complex, which then serve as the focal point for assembly of the complete daughter cytoskeletal and membrane scaffold [2,7].

The structure of the conoid/apical complex is quite remarkable (Figure 1C). The conoid itself is built around a core of tubulin [8,9] arranged into a unique ribbon-like polymer that is quite different from conventional MT [10]. The T. gondii tubulins are unlikely to be solely responsible for the novel structure of this tubulin polymer, because the major αβ-tubulins in *T. gondii* share ~90% sequence identity with mammalian tubulins, and they are incorporated into not only the conoid fibers but also the conventional MT, such as the two intraconoid and the 22 subpellicular MT. Therefore, the specialized arrangement of tubulin in the conoid fibers is probably determined by association with other nontubulin proteins. It is reasonable to expect that an organelle of the size of the conoid (~0.3 × 0.4 μm) would include hundreds of different protein species, but none has yet been identified.

T. gondii and the other Apicomplexans are important pathogens for which better therapy is sorely needed. Their specialized apical complex provides functions that underlie both host-cell invasion and daughter parasite formation, the underpinnings of their pathogenicity, and deserves study for this reason alone. Beyond that, the unique structure of the conoid/apical complex also promises insight into problems that concern all cell biologists, such as the assembly of macromolecular machines based on tubulin. Progress toward these goals has been severely hampered by the almost complete lack of knowledge of the proteins making up the apical complex. In this study, we report: 1) a partial purification of the cytoskeleton of the apical complex; 2) the identification of most of its protein components; and 3) characterization of six new apical complex proteins and analysis of their incorporation into developing daughter parasites.

## Results

### Isolation of the Conoid/Apical Complex Cytoskeleton

**Figure 2:**
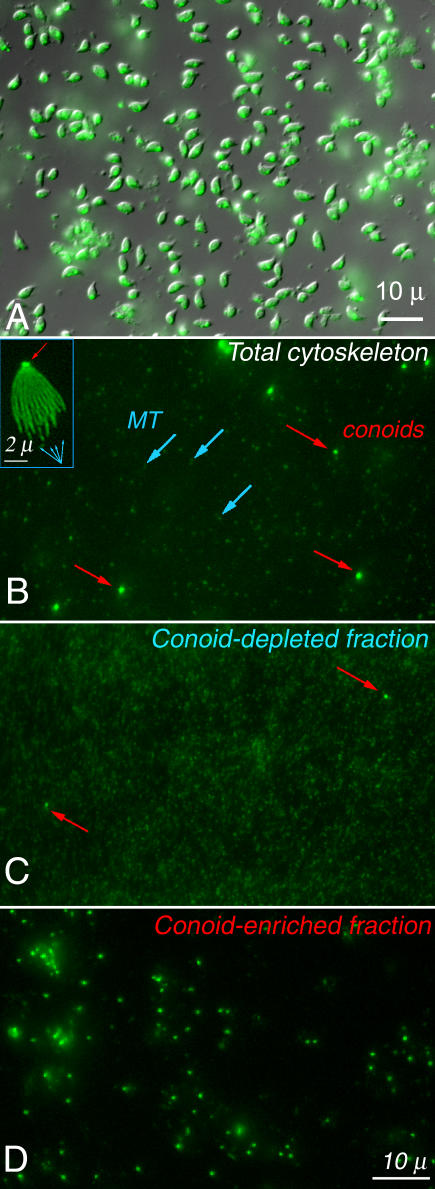
Isolation of the Conoid/Apical Complex (A) Combined DIC and epifluorescence image of the starting material, a suspension of extracellular YFP-α-tubulin (green) transgenic parasites. (B) Epifluorescence image of detergent extracted, sonicated parasites. The fluorescent material, a small fraction of the total mass, is composed of small MT fragments (cyan arrows) and conoids (red arrows). The inset shows an intact “ghost,” a detergent extracted parasite, at 2× higher magnification. The total cytoskeleton prep was fractionated by differential centrifugation into the conoid-depleted (C) and conoid-enriched (D) fractions.

Extraction of T. gondii with detergents leaves a ghost consisting of apical complex, attached subpellicular MT [11], and cortical filament network [12] enclosing fragments of other subcellular organelles. Sonication or other mechanical stresses shear off the apical complex components from the rest of the cytoskeleton, releasing entrapped fragments of other organelles. After vigorous detergent extraction and sonication, the conoids remain intact, recognizable as bright fluorescent dots when prepared from T. gondii expressing YFP-α-tubulin (Figure 2, red arrows), whereas the subpellicular MT are fragmented and much dimmer (Figure 2, cyan arrows). The conoid and remnants of the apical complex cytoskeleton are easily separated from the fragmented MT by differential centrifugation. Figure 2D shows the most rapidly sedimenting fraction, containing ~90% of the conoids, and Figure 2C shows the slowly sedimenting fraction, containing subpellicular MT with very few conoids. Analysis of the fractions by epifluorescence microscopy (to count the number of conoids) and total protein assay gave an estimated enrichment of ~500-fold compared with intact cells, or 50-fold relative to detergent extracted ghosts (Table 1).

**Table 1:**
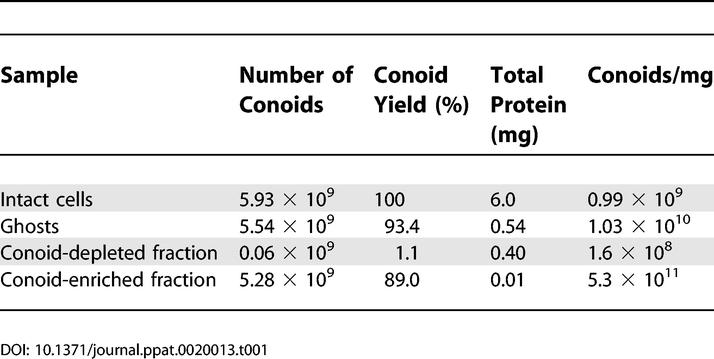
Partial Purification of the Conoid/Apical Complex Cytoskeleton from T. gondii Expressing YFP-α-Tubulin

**Figure 3:**
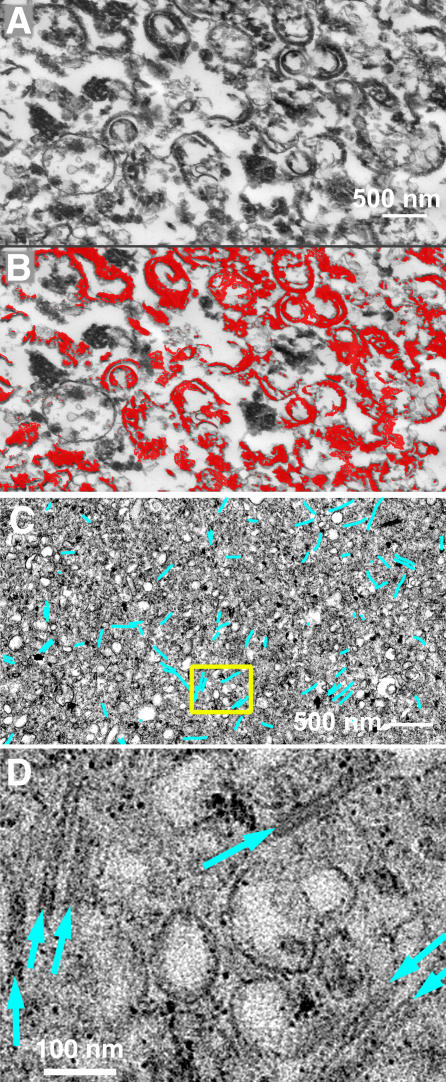
EM Analysis of the Conoid-Enriched and Conoid-Depleted Fractions (A) EM image of a thin section through a pellet of the centrifuged conoid-enriched fraction. (B) To give a rough visual estimate of the enrichment, material that is recognizably fragments of the conoid + polar rings has been colored red (C). (C) EM analysis of the conoid-depleted fraction; MT are marked with cyan lines, which are much wider than the image of the MT at this magnification. (D) Enlarged view of the region within the yellow box, showing small vesicles and MT (cyan arrows).

Compact pellets, formed by resedimenting each fraction in an ultracentrifuge, were fixed, sectioned, and examined by EM as shown in Figure 3. Most of the material in the conoid-enriched fraction is recognizable as conoid/apical complex cytoskeleton sectioned in random orientations (Figure 3A and 3B; red mask). The high-speed pellet of the conoid-depleted fraction shows numerous MT (Figure 3C and 3D, cyan mask) interspersed among tiny vesicles reminiscent of a microsome preparation from a typical eukaryotic cell.

### Identification of Candidate Apical Complex Proteins by MudPIT

The protein composition of the conoid-enriched and conoid-depleted material was determined by the Multidimensional Protein Identification Technology (MudPIT) protocol [13,14]. The mass spectra were analyzed by modified SEQUEST software [15] to give the most probable assignment of each spectrum to a peptide with amino acid sequence corresponding to a segment of an open reading frame (ORF) in the essentially complete T. gondii genome sequence [17]. ~770,000 peptide-spectra pairs were filtered to exclude ambiguous profiles, and the survivors (~72,000) were subjected to a second round of screening (see Materials and Methods) using a discriminant function [16] to select only the most reliably identified peptides (46,981 spectra; 8954 peptides). Peptides corresponding to overlapping segments from a single ORF were assembled into a nonredundant set of 5022 contigs, which were matched against the complete set of predicted [17] and known T. gondii proteins. Of the 1157 proteins identified in at least one of the ten to 12 replicate analyses of each fraction (see Datasets S1–S7 for a complete listing of all components), ~ 30% are unique to one or the other fraction (Table 2). A significant minority (~35%) of the proteins are readily recognized as contaminants from other subcellular organelles. For example, the conoid-enriched fraction includes ~95 recognized ribosomal and ~60 mitochondrial proteins. Of the 286 proteins present in the conoid-enriched fraction only, 23 are known *Toxoplasma* proteins, 53 are unknown proteins with a conserved domain identified in other organisms, 47 unknown proteins with homologues in *Plasmodium* only, 57 unknown proteins recognized only in *T. gondii,* and 107 identified proteins from other organelles. In addition, peptides were detected from 194 genomic ORFs not predicted to belong to a protein by any of the gene models for T. gondii. Of these peptides, 111 have homologues in *Plasmodium* and are thus likely to represent genuine proteins. Most of these unattributed peptides probably identify errors in positioning of exon–intron boundaries by the gene-finder algorithms, since neighboring stretches of amino acids within the same ORFs are usually correctly predicted to be protein coding sequences. Statistical analysis (see Materials and Methods) of the frequency with which individual proteins/ORFs were missed in replicate analyses indicates that the identified components constitute ~70% of the total number of proteins present.

**Table 2:**
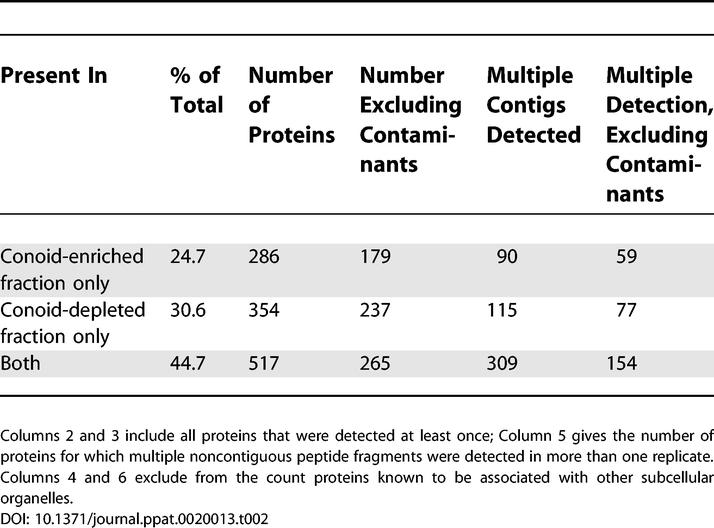
Distribution of 1157 Proteins Identified by MudPIT among 10–12 Replicate Analyses of Cytoskeletal Fractions of T. gondii

As expected, the list of identified proteins includes many cytoskeletal proteins known to be present in the apical complex, including actin, actin-binding proteins, and both heavy and light chains from several different types of myosin [18–24]. Tubulin is abundant in both the conoid-enriched and conoid-depleted fractions. Interestingly, three different isoforms of ß-tubulin were detected, (57.m00003, 41.m00036, 28.m00301), though T. gondii was believed to have only one α- and one ß-tubulin gene [25]. The three isoforms are >96% identical, differing significantly only in the C-terminal ~15 aa, which is the primary isoform-specific region in typical eukaryotic αβ-tubulins. *Eimeria* and *Plasmodium* spp. also have the same three nearly identical β-tubulins.

### Localization of Candidate Apical Complex Proteins by Light and Electron Microscopy

After excluding proteins from other organelles, 59 proteins identified by multiple peptides in multiple replicates (Table 2) were found exclusively in the conoid-enriched fraction. These are good candidates for proteins restricted to the conoid/apical complex in intact parasites. One unidentified protein found predominantly in the conoid-depleted fraction, and six previously unidentified proteins found predominantly or exclusively in the conoid-enriched fraction, were cloned and expressed as fluorescent fusion proteins in transgenic parasites. The numbers following each protein name are at the Draft 3 Annotation gene numbers, as listed at http://toxodb.org/cgi-bin/gbrowse/gbtoxo_31.

#### A new IMC family member (44.m00031).

**Figure 4:**
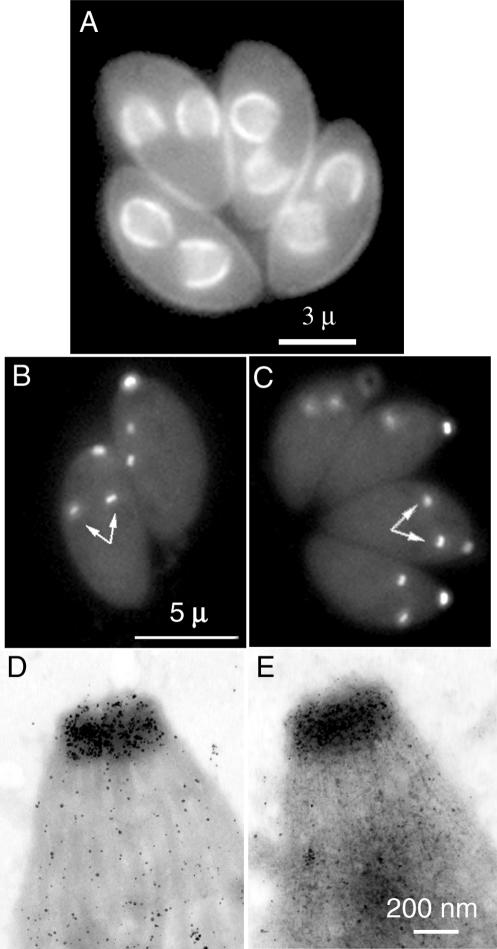
Localization of T. gondii IMC4, TgCAM1, and TgCAM2 (A) LM localization of a T. gondii protein (TgIMC4) with weak similarity to articulin family members, and weaker similarity to TgIMC1. Fluorescence LM images of living transgenic parasites expressing TgIMC4 fused to the C-terminus of EGFP. Fluorescence is observed on both the mother and daughter IMC. FRAP reveals significant differences in the turnover of TgIMC1 and TgIMC4 (unpublished data). (B and C) Fluorescence LM images of living transgenic parasites expressing GFP-CAM1 (B) and GFP-CAM2 (C). Bright fluorescence is observed in the conoid region of both mother and daughters (arrows). (D and E) EM images of T. gondii cytoskeletons from the same two lines of transgenic parasites as in (B) and (C [immunogold-labeled with anti-GFP antibody and negatively stained with phosphotungstic acid]). Specific staining occurs over the conoid itself, and lightly over the subpellicular MTs. In (D), diagonal lines of gold particles are visible, tracing the conoid fibers.

The first of the seven proteins in this pilot project is an unknown protein, present in both fractions but predominantly in the conoid-depleted fraction, with weak homology to the articulin family of membrane skeletal proteins and to IMC1 [12,26]. We chose this protein for two reasons. First, because of the weak homology to the articulin family of membrane-skeletal proteins, and second, our data predicted that the protein would be cytoskeletal but not in the conoid, so we saw this as a negative control to validate our assignment of conoid localizations to the other six proteins described below. The EGFP-tagged protein has a distribution (Figure 4A) similar to IMC1, outlining the inner membrane complex (IMC). However, FRAP analysis (unpublished data) suggests that maternal protein may be recycled into daughters, unlike IMC1 [26,27]. Dr. Con Beckers has also identified and cloned this protein from T. gondii (unpublished data), and designated it IMC4, a new member of the family of IMC structural proteins.

#### T. gondii putative calcium-binding proteins 1 and 2 (TgCAM1 and CAM2; 50.m03141 and 55.m04968).

TgCAM1 and TgCAM2 are two previously unidentified proteins, each with two EF-hand calcium-binding domains, but with different arrangements of the EF-hands within their primary sequence, and with no significant sequence similarity to each other or to other known proteins outside their calcium-binding domain. We chose them because we found their peptide fragments in the conoid-enriched fraction only, and the protrusion of the conoid was known to be responsive to calcium-ionophore treatment. Both TgCAM1 (Figure 4B and 4D) and TgCAM2 (Figure 4C and 4E) localize to the conoid by light and electron microscopy. EGFP-TgCAM1 remains attached after deoxycholate treatment, whereas much of the EGFP-TgCAM2 is removed (unpublished data). Ca^2+^ plus Ca^2+^-ionophore treatment has no effect on the conoid localization of either protein, nor on their extractability by deoxycholate (unpublished data).

#### T. gondii dynein light chain (TgDLC; 41.m01383).

**Figure 5:**
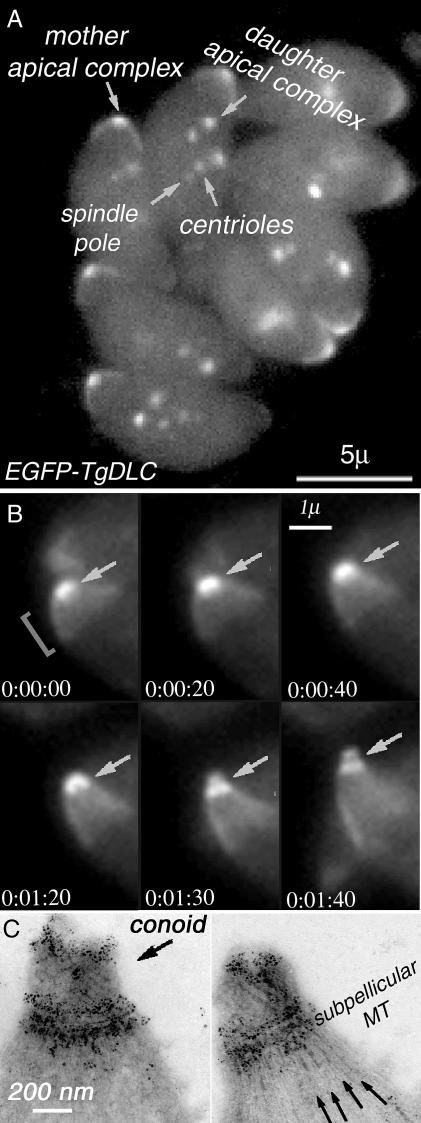
LM and EM Localization of T. gondii Dynein (A) Fluorescence LM image of a vacuole containing eight living transgenic parasites expressing EGFP-DLC. Bright fluorescence is observed in the conoid region of both mother and daughters and in an apical cap. See bracket in (B), in centrioles, and in spindle poles; arrows are identification of the centrioles, and spindle poles is based on colocalization with centrin and tubulin [7,9]. (B) Time-lapse imaging of live EGFP-TgDLC transgenic parasites. Elapsed time after addition of the Ca^2+^ ionophore A23187 is shown at the bottom of each frame. The bright spot of EGFP at the apical end of the parasite (arrow) elongates as the conoid protrudes through the polar ring in response to the elevated intracellular [Ca^2+^] (Video S1). After conoid protrusion, the fluorescence is seen to be concentrated in two bands of unequal width. The entire apical end of the cell narrows and elongates at the same time. (C) Two EM images of Ca^2+^ ionophore–treated, deoxycholate-extracted EGFP–TgDLC transgenic parasites, immunogold-labeled with anti-GFP antibody and negatively stained with phosphotungstic acid. Numerous gold particles are located on the apical and basal ends of the conoid fibers as well as on the polar ring at the sites of attachment of the subpellicular MT, but are sparse along the length of the fibers and MTs, recapitulating the distribution of fluorescence in (B).

T. gondii dynein light chain (TgDLC) is a small (8-kD) protein, >85% identical to DLCs in mouse and human. We selected this protein because the prominent role played by tubulin in conoid structure naturally prompts one to look for MT-dependent motor proteins*.* By light microscopy (Figure 5), EGFP-TgDLC localizes in spindle poles and centrioles, in a bright spot at the apical end of the parasite, in an apical cap extending down the periphery of the parasite for ~ 1.5 μm, and in a small ring at the basal end of the parasite. The apical cap extends for a much shorter distance than do the subpellicular MT. The apical spot was identified as the conoid by observing the extension of the brightly fluorescent apical end after Ca^2+^ ionophore treatment [5] (Video S1). The distribution of EGFP fluorescence across the extended apical spot is not uniform; rather, it appears as two bands with dark space in between (Figure 5B, 1-min–40-s frame). This intensity distribution correlates exactly with higher resolution localization by immuno-EM (Figure 5C), revealing specific localization to the two ends of the conoid fibers as well as at the sites of attachment of subpellicular MT to the polar ring, the (−)end of the subpellicular MT [28]. This suggests that dynein (a (−)end-directed motor [29]) may transport cargo along these tracks. Apart from this specific end-labeling, EGFP-TgDLC is bound only sparsely along the length of the subpellicular MT and conoid fibers, suggesting that the observed pattern is not nonspecific binding due to overexpression.

In Figure 5B, the apical cap of fluorescence appears to elongate as the cell becomes narrower. How much of this is real elongation and how much is due to other effects (decreased foreshortening due to changes in view angle; straightening of curved fibers to which dynein is bound) has not yet been ascertained.

#### T. gondii MORN-domain–containing protein (TgMORN1; 583.m05359).

**Figure 6:**
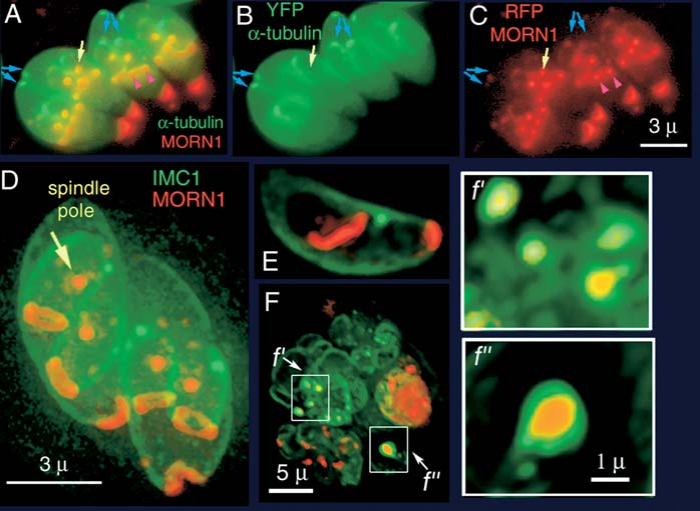
LM Localization of a T. gondii MORN Domain Protein, TgMORN1 (A–C) Single optical section of living transgenic parasites expressing YFP-α-tubulin (green) and mRFP-TgMORN1 (red). Bright red fluorescence is observed at the spindle poles (yellow arrows), near the ends of the growing IMC in developing daughters (pink arrowheads), and in a conical patch at the basal end of the mature parasite. Weaker fluorescence is observed in the region of the conoids of both mother and daughters (blue arrows). Note in (B), showing YFP-tubulin only, that two small green dots are seen in each daughter. The more apical dot is the centriole, the more basal dot (yellow arrow) is the spindle pole [7,9]. (C) TgMORN1 accumulates at the basal dot (spindle pole, yellow arrow) but not the centriole. (D–F) Double transgenic parasites expressing IMC1-YFP (green) and mRFP-TgMORN1 (red). (D) Projection of a deconvolved 3D stack of images. MORN1 is clearly seen to form a ring around the basal end of the developing daughter scaffold. (E) Projection of a deconvolved 3D stack of images of an extracellular parasite, showing a cytoplasmic fiber of TgMORN1. No daughters are present. Same scale as (D). (F) Parasite culture treated with oryzalin for 24 h before imaging. A vacuole containing four grossly distorted parasites. IMC1 accumulates as large shells and smaller blobs. *f′* & *f′′:* higher magnification views (rotated 90°) of IMC1 blobs from (F) revealing a core of TgMORN1.

This protein was selected initially by a serendipitous confusion that misled us to think it might be related to an interesting kinase. In fact it has no kinase domain, but proved to be interesting nonetheless for other reasons. TgMORN1 is a ~41kDa protein composed almost entirely of a MORN (membrane occupation and recognition nexus) domain. The MORN domain is conserved from bacteria to mammals, but its function is unknown. It is found in proteins of variable size, being composed of variable numbers of 23 aa repeats. The best studied MORN-containing proteins are the junctophilin family, involved in membrane-cytoskeletal interaction [30]. TgMORN1 contains 14 repeats of the conserved 23aa motif. EGFP- or mRFP-tagged TgMORN1 labels the conoid, but its predominant localization is in the form of a cap at the basal end of both mature mothers and developing daughter parasites (Figure 6). In addition, TgMORN1 also shows strong fluorescence at the spindle poles and in a weaker diffuse cloud around the spindle poles. Deconvolved 3D images (Figure 6D) reveal that the basal accumulation in the daughters is actually a ring encircling the basal end of the developing daughter IMC scaffold. In the adult, TgMORN1 fills in the gap at the basal end of the parasite that is not occupied by the IMC. Although MORN domains are thought to somehow provide an interaction between membranes and the cytoskeleton, their primary sequence gives no hint of a transmembrane or intramembrane localization. In accord with this, TgMORN1 distribution is unaffected by treatment with nonionic (Triton X-100, Tween 20) or ionic (deoxycholate, CHAPS) detergents (unpublished data).

The ring of TgMORN1 at the end of the growing daughter scaffold could be related to the growing ends of subpellicular MT. For comparison with TgMORN1, we expressed in T. gondii a mRFP-tagged mammalian EB1 protein, which binds to the (+)ends of dynamic MT [31]. EB1 (no sequence homology to TgMORN1) localized in the spindle pole and near the tips of the growing daughter subpellicular MT, but with a more diffuse appearance than TgMORN1, and did not appear in the conoid (unpublished data). As an incidental result, these experiments also provide independent experimental confirmation of the orientation of the subpellicular MT in T. gondii [28].

To correlate the biogenesis of the basal TgMORN1cap with construction of daughter scaffolds, we treated T. gondii with the MT-destabilizing drug oryzalin, which blocks cytokinesis while many other processes associated with daughter formation continue. In particular, the cytoplasm of the large rounded polyploid cells becomes filled with disorganized IMC [11,32–34]. Accumulations of IMC1 occur in sheets and sometimes as small blobs (Figure 6F). Interestingly, many of these small IMC1 blobs were found to have a core of MORN1 (Figure 6F), again suggesting TgMORN1 involvement in assembly of the IMC, perhaps as a nucleator rather than a capper.

Among extracellular parasites that have remained outside a host cell for several hours, a few develop large cytoplasmic fibers of TgMORN1 (Figure 6E). These fibers are ~0.5 μm wide and variable in length, but may be quite long, >5 μm, and, when long, tend to coil around the nucleus. The position, shape, and length vary from cell to cell. These are most likely to be aggregates caused by overexpression of the fusion protein, but it is interesting that the aggregates develop in this highly asymmetric, presumably paracrystalline, fibrous shape, possibly reflecting the tendency of the MORN domain to self-assemble.

#### TgCentrin-2 and TgCentrin-3 (50.m03356 and 55.m00143).

**Figure 7:**
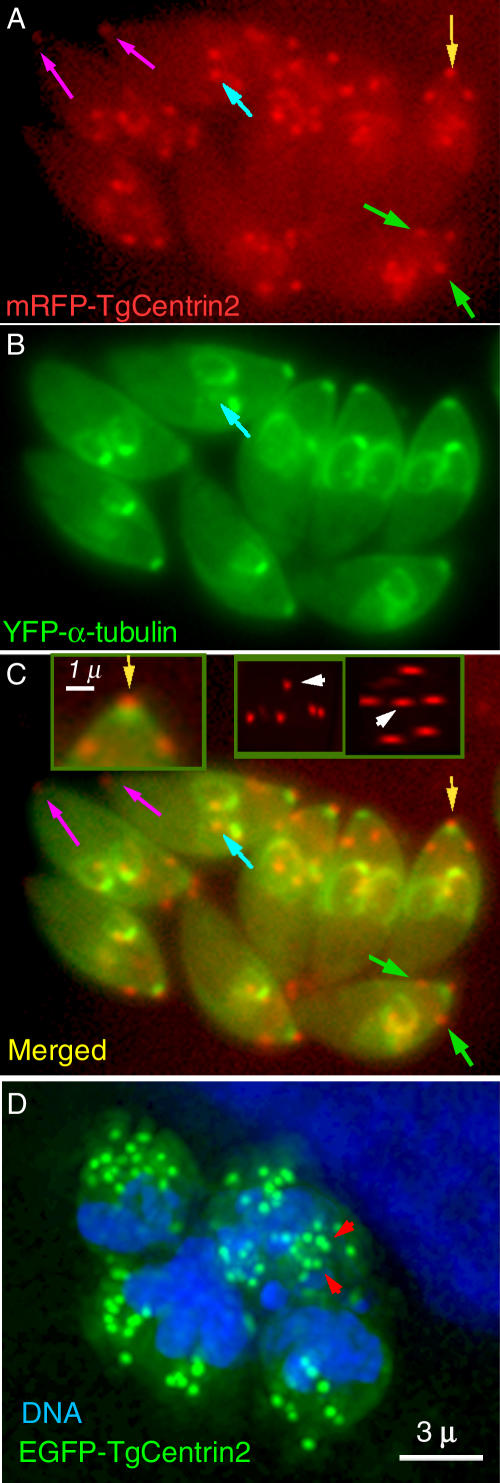
Distribution of T. gondii Centrin-2 (A–C) Fluorescence LM images of a vacuole containing eight living transgenic parasites expressing mRFP-centrin-2 and YFP-α-tubulin. Bright fluorescence is observed in the extreme apical region (yellow arrows), centrioles (blue arrows), five spots around the circumference near the apical end (green arrows; only two or three are visible in one focal plane with intact parasites), and a single small patch at the basal end (magenta arrows). The left inset in (C) shows an enlarged view of the apical region. The pair of smaller insets show two projected views of a deconvolved 3D stack of images, looking from the side (left) and from the apical end (right) of a single parasite. White arrowheads point to the apical spot; five other spots are organized in a ring around the circumference of the parasite. (D) Projection of a 3D stack of images of a vacuole containing four parasites from a transgenic parasite line expressing T. gondii EGFP-centrin-2 (green), after 24 h of oryzalin treatment. DNA (blue) is visualized with the cell-permeant dye Hoechst 33342. A host cell nucleus abutting the parasitophorous vacuole (upper right corner of the image) has been digitally attenuated for clarity. Clusters of EGFP-TgCentrin2 dots are dispersed throughout the cells. A minority of the dots occur as closely spaced pairs, presumably daughter centrioles (red arrowheads).

**Figure 8:**
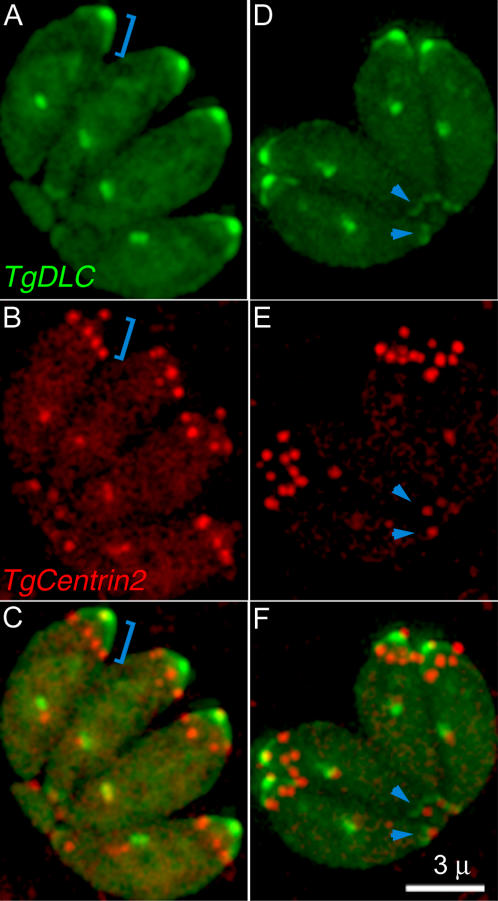
Colocalization of T. gondii Centrin-2 and DLC Fluorescence images (projections of a deconvolved 3D stack) of two different vacuoles (A–C) (D–F), each containing four parasites expressing both EGFP–TgDLC and mRFP–TgCentrin2. Blue brackets in (A–C) mark the position of the apical cap of dynein. Note that the ring of TgCentrin2 spots is always positioned at the lower border of this cap. The arrowheads in (D–F) indicate the faint basal ring of dynein that lies adjacent to the basal spot of centrin2.

**Figure 9:**
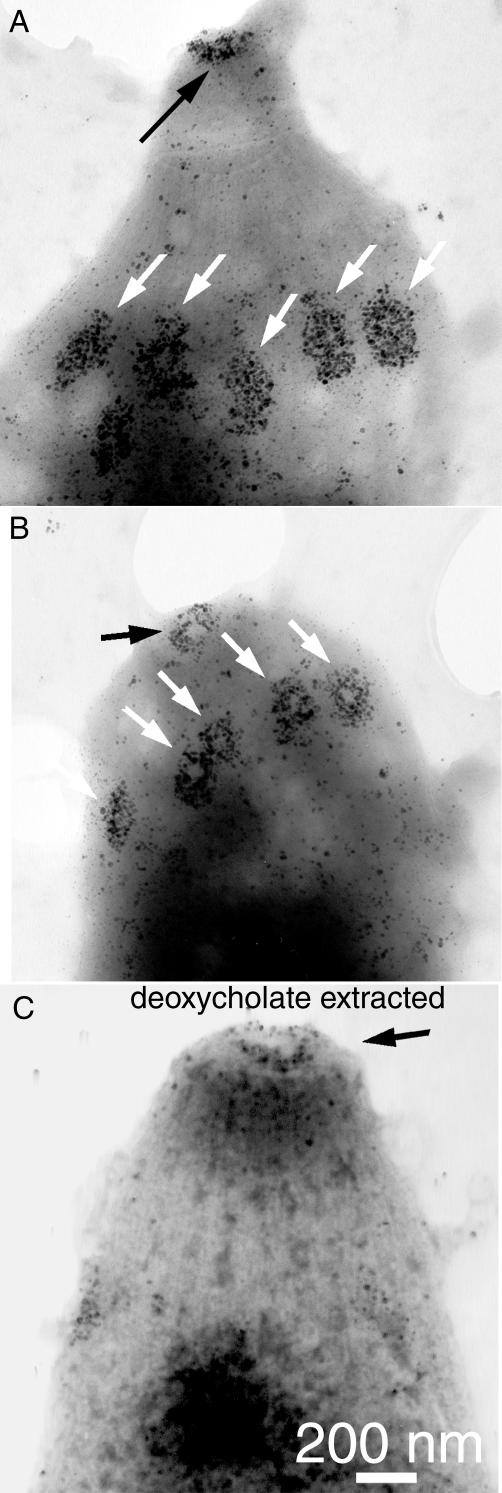
Immuno-EM Localization of T. gondii Centrin-2 (A–B) Two EM images of Triton-extracted cytoskeletons from a transgenic parasite line expressing T. gondii EGFP–centrin-2, immunogold-labeled with anti-GFP antibody, and negatively stained with phosphotungstic acid. Specific staining is seen at the preconoidal rings (black arrows), and in five ~200nm annular patches (white arrows). After deoxycholate extraction (C), the annular patches disappear almost completely but the preconoidal ring staining remains. The large dark blob in the center of the parasites is primarily attributable to accumulation of the phosphotungstic acid in thicker regions, but may also include some cytoplasmic and centriolar concentrations of centrin-2.

Two centrin homologues were detected; TgCentrin2 predominantly in, and TgCentrin3 exclusively in, the conoid-enriched fraction. Both are highly homologous (~60% identical aa) to the previously cloned TgCentrin1 [7]. Comparison with centrin family members from other organisms shows that TgCentrin3 is the most conserved of the three centrins in T. gondii (>60% identical to the consensus sequence), and TgCentrin2 is significantly more divergent (~40% identity). We selected these proteins because in other cells centrins are known to be associated with the centrosome and MT organizing center [35,36]. EGFP-TgCentrin1 localizes to the centriole only [7], whereas EGFP-TgCentrin3 is found in the centriole, but in addition shows faint localization in the conoid (unpublished data). In accord with its more divergent primary sequence, EGFP-TgCentrin2 shows a dramatically different localization (Figure 7). In addition to diffuse cytoplasmic and bright centriolar fluorescence, highly localized patches of TgCentrin2 are seen at the extreme apical and basal ends of the parasite, and in a ring of five to six small patches around the circumference ~1.5 μm from the apical end of the cell (Figure 7). In transgenic parasites expressing both Centrin2 and TgDLC, the ring of TgCentrin2 always coincides with the lower edge of the apical cap of dynein described above (Figure 8). A smaller and weaker basal cap of dynein is also seen surrounding the basal spot of centrin2. EM images (Figure 9) of immunogold-labeled parasites show that the apical circumferential patches are in fact annuli (the central clear space is seen only when the direction of view is appropriate, as in Figure 9B). EM images also reveal that the extreme apical-end–labeling is confined to the preconoidal rings. We have not yet been able to determine the detailed arrangement of the TgCentrin2 accumulation at the basal end of the parasites because the ultrastructure at that end is destroyed by detergent treatment (necessary to allow penetration of the gold-conjugated antibody for immunoEM), perhaps due to the lack of support from the subpellicular MT. The polar-ring–labeling is resistant to extraction by deoxycholate, but the labeling on the circumferential patches is greatly reduced (Figure 9C).

After 18–24 h of oryzalin treatment, TgCentrin2 is no longer arranged as an organized peripheral necklace, but is present as clusters of bright dots throughout the cell (Figure 7D). Beyond a tendency for some dots to occur in pairs (arrowheads, Figure 7D), there is no obvious pattern of the centrin clusters, which contain two to ten centrin dots separated from each other by ~1 μm. These clusters do not seem to associate with either accumulations of IMC1, or lobes of the polyploid nucleus.

### Construction of the Apical Complex

**Figure 10:**
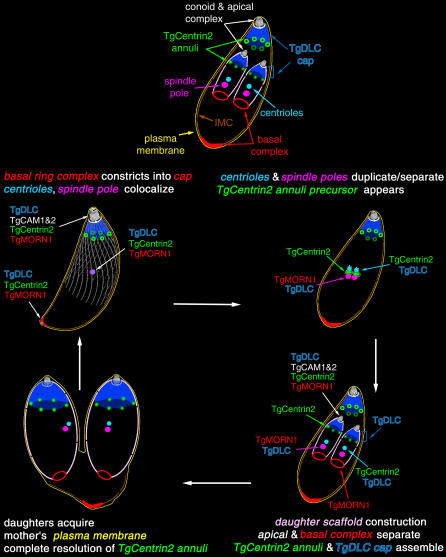
Time-Lapse Image Imaging of Incorporation of YFP-α-Tubulin (Green) and mRFP-TgCentrin2 (Red) into Developing Daughter Parasites Combined DIC and fluorescence images are shown for the beginning (*t* = 0) and end (*t* = 250 min) of the sequence, during which a vacuole of four parasites progresses from the onset of mitosis through completion of budding out of the eight daughters. See text for a detailed description of the sequence of events.

**Figure 11:**
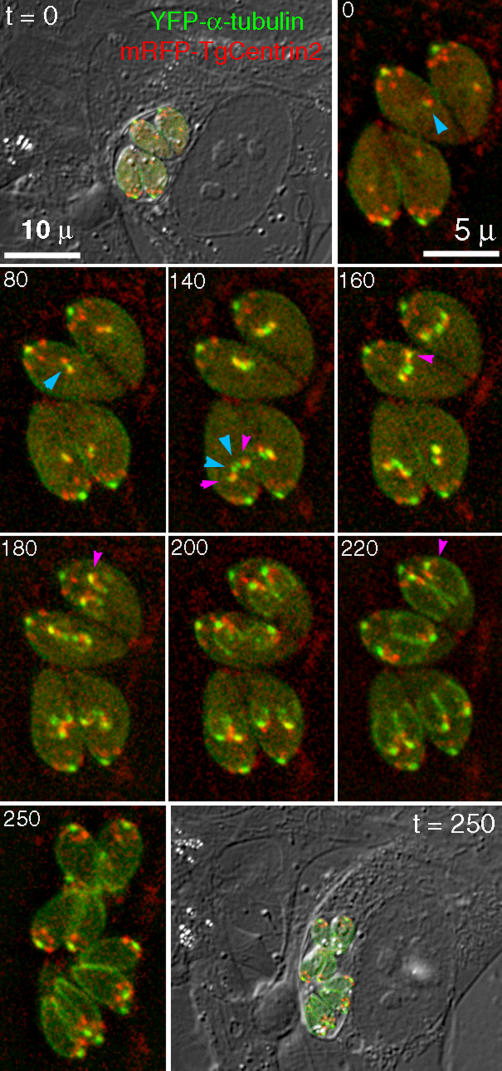
Cartoon Diagrams of T. gondii at Various Points during Its Cell Cycle, Summarizing the Time-Course of Incorporation of the Proteins Discussed in the Text (Upper) The major structural reference points are labeled in a cell containing two growing daughters. The maternal IMC is shown in brown. The unlabeled daughter IMC/scaffold is shown in pink. (Middle and Lower) The cell cycle is traversed starting with an interphase adult cell at the top left, and proceeding clockwise through early, mid, and late stages of daughter formation. The remnants of the mother are left behind as a “residual body” when the daughters bud out (lower left). The variable color scheme for the organelles is intended to emphasize colocalization (e.g*.*, green Centrin2 + blue DLC = cyan; red MORN1 + blue DLC = magenta; multiple color overlaps are shown in white). Subpellicular MTs are always present in mothers and in daughters from very early on, but are shown only for the interphase adult cell.

Having identified these new apical complex components, we can now use them to study dynamic aspects of conoid/apical complex cytoskeleton assembly (see Figure 11 for a cartoon schematic overview of the events described below). What are the very early processes that provide a template for the final structure, determine its striking polarity, and might provide clinically relevant targets for disruption of parasite replication? When and where do these early steps occur? Centriolar duplication currently provides the earliest observable event associated with daughter cell formation in *T. gondii,* as for mitotic division in other systems [35,37–39]. Daughter conoid formation is initiated in close apposition to the recently duplicated centrioles [7]. In YFP-α-tubulin parasites, these events are manifested as an increase in YFP fluorescence near the centriole, before the daughter centrioles separate. Figure 10 shows a sequence of images from parasites expressing both YFP-α-tubulin (green) and mRFP-TgCentrin-2 (red). In interphase, TgCentrin2 colocalizes with YFP-α-tubulin at the centriole (blue arrowhead, time 0). At the beginning of cell division, the mRFP signal splits into two dots and the YFP-α-tubulin signal elongates (80 min, blue arrowhead), as the duplicated centrioles separate and the spindle poles form in between. An hour later, the YFP-α-tubulin signal resolves into four spots, a pair of spots in the middle (two spindle poles, unresolved in some cells at this point; *t* = 140, blue arrowheads), and two bright spots on the side (magenta arrowheads) that overlap partially with prominent TgCentrin2-labeling. These outer spots include overlapped conoidal and centriolar YFP-α-tubulin, as the daughter conoid formation initiates around the centrioles [7].

Separation of the red TgCentrin2 and green YFP-tubulin fluorescence at the centriole starts to become visible at ~140–160 min. Extra TgCentrin2 fluorescence surrounds the dots of YFP-α-tubulin–labeled centriole/conoid (*t* = 160; magenta arrowhead). The conoid becomes distinct from the centriole as the arch of the growing daughter scaffolds (outlined by subpellicular MT) becomes first discernible at ~1.5 h after separation of the daughter centrioles. By this time, TgCentrin2 is clearly incorporated into the daughter apical complex (*t* = 180; magenta arrowhead). Some TgCentrin2 fluorescence colocalizes with YFP-α-tubulin at the centriole. Between the apical complex and the centrioles, additional mRFP-TgCentrin2 fluorescence is seen, precursor of the peripheral annuli present in the adult parasites. By 220 min, the peripheral scaffold localization of TgCentrin2 becomes very obvious (magenta arrowhead), and by the end of cell division (*t* = 250) has resolved into the 5–6 distinct dots seen in adult parasites.

In mature parasites in interphase, TgMORN1 is continuously present as a basal cap and at the spindle pole in abundance, as well as in a diffuse cloud surrounding the centrioles and spindle pole. Time-lapse imaging of parasites coexpressing mRFP-TgMORN1 and YFP-α-tubulin or IMC1-YFP reveal that both the developing daughter apical complex and the basal ring capping the daughter scaffold form within this cloud (Video S2; Figure 11). This is quite a surprising result, revealing that the future extreme apical and extreme basal components of this highly polarized cell are assembled at the same time and in the same location, very early in daughter cell formation.

The ring at the basal end of the daughter IMC moves away from the apical complex as the daughter cytoskeletal scaffolds elongate (Video S2). During this period of rapid growth, the diffuse cytoplasmic cloud of TgMORN1 becomes much fainter, and soon a weak accumulation of TgMORN1 in the daughter conoids can be discerned. This apical spot persists as the daughters bud out from the mother, and remains visible in the adult throughout interphase (Figure 6C). TgMORN1 remains as a ring at the terminus of the daughter IMC until after the daughters bud out of the mother, migrating distally to form the basal cap ~15–30 min after budding. The mechanism of this distal migration is particularly intriguing given the presence of concentrated TgDLC and TgCentrin2 (Figure 8D and 8F) but absence of known cytoskeletal elements that could serve as tracks in the extreme basal region of the parasite.

Five additional double-transgenic parasite lines were constructed to study the timing of recruitment of other proteins into the apical complex (unpublished data). Time-lapse imaging of the assembly dynamics of mRFP-tagged TgDLC, TgCAM1, and TgCAM2 were observed when coexpressed with YFP-α-tubulin or IMC1-YFP. Incorporation of these proteins into the apical complex occurs slightly later than TgCentrin2 or TgMORN1. Association of TgDLC, TgCAM1, and TgCAM2 with the apical complex and the daughter scaffolds becomes clearly visible when the scaffolds become resolvable from the conoid 1.5 h after separation of the centrioles (Figure 11).

## Discussion

The cytoskeleton of the apical complex is potentially a prime drug target for inhibiting both parasite replication and invasion into the host cell. Knowledge of its molecular composition is however virtually nonexistent. In this study, we have partially purified the conoid/apical complex cytoskeleton, identified most of its protein components, characterized seven novel proteins, and determined the sequence of recruitment of five into the daughter cytoskeleton during cell division. These results provide new markers for the different subcompartments within the apical complex, and reveal previously unknown cellular compartments, enhancing our understanding of how the invasion machinery is built.

Ideally, one would prefer to have a completely pure preparation of a subcellular organelle before determining its protein composition, but in practice that may be difficult, prohibitively time-consuming, or even impossible for many organelles. The alternative approach adopted here, using a highly enriched but not completely pure preparation in combination with subtractive screening succeeded extremely well. The conoid can be partially purified via a very simple procedure due to its high density. The final preparation is not completely homogeneous, but well within the range of analysis by MudPIT, which has proved capable of identifying many thousands of proteins in much more complex mixtures [14,40]. The primary output from this analysis is a list of MS spectra assigned to peptide fragments of an open reading frame in the T. gondii genome. The identification of peptides by MS/MS in a complex mixture is subject to error [41,42]. This was dealt with by extensive statistical analysis, as described in Materials and Methods.

### How Many Proteins Make Up the Apical Complex?

The efficiency of peptide identification by MudPIT is low, but the efficiency in terms of protein detection is substantially better, since each protein gives rise to many peptide fragments. Even so, a significant fraction (30%) of proteins was confidently identified in only one of the replicate analyses. Such proteins could have been present also in the other fraction but simply failed to show up by chance, leading to overestimation of the proportion of fraction-specific proteins. On the other hand, proteins shared by both fractions might tend to be relatively abundant (as suggested by Table 2), thus more likely to be detected, which would lead to an underestimation of the fraction-specific species. Examples of both of these forms of bias have been reported in experimental data [43].

Statistical methods for treating this kind of intersample variation have been developed for several disciplines [44–47]. Capture–recapture analysis of the incidence (proportion of replicates in which a component was detected) and abundance (number of spectra per component in a single replicate) data suggests that the list of observed proteins accounts for ~70% of the proteins that could have been detected by exhaustive MudPIT analysis. The analysis also suggests that the observed data slightly underestimates the proportion of proteins found only in the conoid-enriched fraction (observed 25%; predicted 31%) and overestimates the ubiquitous components (observed 45%, predicted 31%). Applying this type of analysis separately to the proteins known to belong to other organelles versus the potentially specific structural proteins of the apical complex, we estimate that the total apical-complex cytoskeleton-specific component probably comprises ~300 ± 30 proteins, of which 196 were actually detected. To place this number in perspective, >250 distinct proteins have been detected in 2D gels of eukaryotic flagellar axonemes [48], and 688 proteins were found in cilia + basal bodies by mass spectrometry analysis [49].

Because the conoid-enriched fraction analyzed by MudPIT was not completely pure, some of the peptides will be “contaminants” derived from other subcellular organelles. Two recent studies reported complementary proteomics analysis of rhoptries [50] and of other nonrhoptry secretory organelles [51] of *T. gondii.* These organelles are located in the apical region of the parasite and are thus likely “contaminants” in our samples. We detected 34 of the 46 identified rhoptry proteins, and 23 of the 76 proteins from mixed secretory vesicles. As expected, the overwhelming majority (50 of 57) were not fraction-specific, but abundantly present in both fractions. Six of the proteins were detected in the conoid-depleted fraction only, and one protein showed up in a single replicate of the conoid-enriched fraction. Many other proteins found with high frequency in both fractions (>70%) are also well-known abundant proteins, including proteins from other organelles (e.g., mitochondria, ribosomes). In contrast, 60% of the proteins detected with high frequency in the conoid-enriched fraction only have no clear homologues in higher eukaryotic genomes, and thus may be components of the Apicomplexan-specific conoid–apical complex. Excluding components of other organelles, 59 proteins were detected in multiple replicates by multiple peptide fragments in the conoid-enriched fraction, but never observed in 12 replicate analyses of the conoid-depleted fraction. Indeed, we cloned six proteins that are detected predominantly or exclusively in the conoid-enriched fraction and all six do in fact originate from the predicted cellular location and several provide valuable markers to different subcompartments within the apical complex, including the preconoidal rings (TgCentrin2), the conoid (TgDLC, TgCAM1, and TgCAM2), and the polar ring (TgDLC). In addition, the distribution of two of these proteins (TgCentrin2 and TgMORN1) revealed major structural features of the T. gondii cytoskeleton that had previously been completely unsuspected. One caveat that must be borne in mind, particularly for these proteins observed to localize to several different compartments, is that we cannot exclude the possibility that minor localization changes could occur as a result of the heterologous promoter used for expression and/or the fusion to the fluorescent protein.

No proteins identified in the MudPIT screen and predicted to be part of the cytoskeleton have turned out not to be associated with the cytoskeleton. What are the chances that the predicted cellular localizations would have turned out to be correct merely by chance? In a global sense, this must be roughly the same as the ratio of proteins in the cytoskeleton of the apical complex to total *Toxoplasma* proteins. Of course, the proteins we selected for cloning all have some feature that might indicate a higher probability of localization to the sites that we are interested in, so a more accurate estimate of the probability would narrow the sample space to contain only *Toxoplasma* proteins with those particular features. For instance, the DLC is a component of a MT-based motor complex. Overall, there are 18 putative dynein components and ten putative kinesins in the predicted *Toxoplasma* proteome. Our data predicts that four of those proteins will be found in the cytoskeleton of the apical complex. We cloned one of the four, and it indeed shows the predicted localization in living cells. The probability of this occurring simply by chance is, very crudely, something like four out of 28, or ~15%. Similar calculations, admittedly crude but nevertheless the best that can be done at present, show that one is unlikely to pick at random a protein from the families of those we have cloned that will specifically localize to the cytoskeleton of the apical complex. The probability of randomly picking six proteins that so localize without a single mistake is very small. The only counterargument would be to claim that a majority of the members of each of these families reside in the apical complex cytoskeleton, but that we simply failed to detect them. However, this claim is incompatible with the best estimate of the probability of detecting bona fide residents, which is ~70%.

Most of the proteins we cloned had some homology to other proteins known to be sometimes associated with the cytoskeleton, but of course this is not sufficient to predict localization specifically to the apical cytoskeleton. What about the 59 totally unknown hypothetical proteins for which there is no known homologue and which were detected in the conoid-enriched fraction only? In this case, a somewhat weaker prediction could be made: the probability of localization to the conoid/apical cytoskeleton might be roughly the same as the ratio of total-proteins-minus-known-contaminants to total proteins detected in the conoid-enriched fraction only (i.e., 179/286, ~60%).

Dynein is a (−)end-directed MT motor. Consistent with observations in other systems, the DLC identified in the conoid-enriched fraction accumulates at the (−)end of subpellicular MT as well as at other sites of MT initiation (Figure 5). Interestingly, it also concentrates in a peripheral apical cap, and labels both ends of the conoid fibers, which hints at a mechanism for the Ca^2+^-induced conoid–apical complex motility with dynein serving as motor. At least four different types of motile behavior occur within the apical complex: transport of secretory vesicles, probably along the pair of intraconoid MT; extension and retraction of the conoid through the polar ring; tilt/twist of the conoid fibers themselves [10], and elongation of the entire parasite. Both actin, previously shown to be localized in the apical complex [18,19,52] and several myosin isoforms were identified in the conoid-enriched fraction, as was an actin depolymerizing factor (see Datasets S1–S7). MT-dependent motility is also likely. In addition to dynein, at least one abundant kinesin-like motor protein was found in the conoid-enriched fraction only.

*T.gondii* was believed to have only a single gene each for α- and β-tubulin, but to our surprise three different β-tubulin isoforms are abundant in both fractions. Only one α-tubulin was detected by mass spectrometry, but, having been alerted to the possibility of multiple isoforms, a search of the T. gondii genome sequence revealed two more previously unsuspected α-tubulin genes. All six isoforms are expressed in various stages of the life cycle of T. gondii (Hu et al., unpublished data), and all have orthologs in the *Plasmodium* and *Eimeria* genomes.

Although it is not yet possible to assign specific motors to individual motions, Ca^2+^-binding proteins specifically localized to the apical complex are likely to be involved in regulating its motion, as the movements of the conoid and conoid fibers are activated by increasing intracellular Ca^2+^ concentration. Both of the calmodulin-like proteins enriched in the apical complex fraction, TgCAM1 and TgCAM2, concentrate at the conoid (Figure 4). TgCAM1 and TgCAM2 both share moderate similarity (24%/43% and 32%/50% identity/similarity) to a previously cloned T. gondii calmodulin [53] that is also found in the apical complex [20]. Interestingly, however, there is no significant sequence similarity between TgCAM1 and TgCAM2. Both proteins are incorporated into the apical complex at the beginning of daughter scaffold formation.

Three centrins have been identified in T. gondii. TgCentrin2, in addition to its centriolar localization, is found in peripheral annuli at the apical portion of the parasite body as well as in the preconoidal rings. Although it is difficult to tell from LM or EM images of adult parasites, these annuli are probably located on the IMC rather than the plasma membrane, as they are well-developed before daughter cells acquire a plasma membrane by budding out from the mother. The assembly and maturation of these peripheral annuli occurs in lock-step with daughter scaffold assembly. Their precursor appears to be a nebula of TgCentrin2 enclosing the centrioles early on during cell division, which later matures into distinct dots arranged in a peripheral ring. In mature cells, the ring of TgCentrin2 annuli is consistently associated with the lower edge of the apical cap of dynein. The number (five to six) and arrangement of these annuli (Figures 7–9 and 10) hints at a role in organizing the IMC, which is formed from a number of flattened vesicles, appearing in freeze fracture images as a patchwork of plates joined together by sutures [32,54,55]. The total number of plates is difficult to determine, but five to six plates spanning the circumference of the parasite is consistent with the available data. Furthermore, a sharp transition between two distinct patterns of intramembrane particles on the IMC plates occurs at approximately the same position on the parasite as the location of the TgCentrin2 annuli and lower edge of the dynein cap, suggesting some functional connection. This cap region was previously shown to harbor a set of apically localized proteins defined by a panel of monoclonal antibodies [32,56].

The primary sequence of TgMORN1 is taken up almost entirely with 14 repeats of the MORN motif, which is found in a diverse set of proteins in all organisms. In *T. gondii,* MORN domains occur in a phosphoinositol kinase, a DNA methyl transferase, a prolyl cis-trans isomerase, and numerous otherwise unrelated proteins. The domain appears to be a widely used building block that has been repeatedly adapted to mediate protein–protein interactions, similar to leucine zippers or other general purpose protein–protein interaction modules. The elongated fibers of EGFP-TgMORN1 (Figure 6E) observed in many extracellular parasites probably result from a propensity for self-association coupled with steady state levels of the fusion protein that are inappropriate for extracellular parasites, in which the cell cycle is arrested.

TgMORN1 concentrates at the conoid and the spindle pole, and it is also the first protein shown to be localized to the basal ends of mother and daughter scaffolds (Figure 6). The extreme basal end of the parasite, unlike most of the parasite body, is not enclosed by the IMC, and it has been unclear whether some other structure occupies the gap. TgMORN1 thus provides a key to understanding how this compartment originates and is constructed. During cell division, a ring of TgMORN1 is incorporated onto the growing basal tips of daughter cells as soon as the daughter scaffolds take shape. The ring of TgMORN1 then descends with the growing terminus of the daughter scaffolds and finally constricts into a solid structure capping the basal end of mature parasites, where it fills in the gap unoccupied by the IMC network (Video S2, Figure 11). TgMORN1 is likely to be one of the components of the electron dense material associated with the tip of the forming daughter scaffold seen in early EM work [57]. The localization of TgMORN1 as a ring at the growing edge of the daughter scaffold and its subsequent collapse into a patch at the basal end is strikingly reminiscent of the rings and bud scars marked by actin and other proteins at the site of budding in yeast [58].

Our time-lapse data show that, as might have been expected, conoid/apical complex cytoskeleton assembly occurs as an ordered sequence of incorporation of different protein components (see Figure 11 for a schematic summary). The proteins we characterized are now available as a set of markers for monitoring this assembly sequence. What was unexpected is that assembly of the future basal components of the parasite occurs at the same time and in the same location as the earliest apical complex assembly. Heretofore no components of the basal end of the parasite had been identified, so TgMORN1 also provides the first structural marker for the extreme basal end of the parasite.

Both temporally and spatially, the initial steps of conoid/apical complex cytoskeleton assembly occur in close proximity to duplication and separation of the centrioles [7,33]. It is also now clear that centrioles, spindle poles, and conoids share other protein components in addition to their core of tubulin. These multiple affinities make it reasonable to ask whether the centrioles provide a templating function for conoid assembly. Consistent with this hypothesis, a homologue of a component of the gamma-ring complex is found in the conoid-enriched fraction (Datasets S1–S7).

Probing the function and assembly of this intriguing but mysterious cytoskeletal compartment, the apical complex, has been impossible due to lack of knowledge of its molecular composition. The protein identifications reported here remove that obstacle, and open the way to systematic development of inhibitors of parasite invasion and replication. Furthermore, as evidenced by three of the seven proteins we described in detail, the list is rich in proteins whose study reveals unsuspected facets of the cell biology of these human pathogens. We are eager to see the remainder of the list explored by all interested investigators as rapidly as possible, and encourage our colleagues to contact us if we can assist in promoting or coordinating that exploration.

## Materials and Methods

### Parasite culture and harvest.

T. gondii tachyzoites (strain RH) were cultivated in human foreskin fibroblast cells as previously described [59]. Parasites were harvested soon after emerging from host cells, passed through a 3-μm filter (Whatman #110612, Whatman, Brentford, Middlesex, United Kingdom), centrifuged at 1000 g for 12 min, washed once with PBS, and pelleted with desktop centrifugation for 3 min. The supernatant was removed and the parasite pellets were either immediately used for purification of the conoid or kept frozen at −80 °C for later use. For some preparations, the parasites were treated with 20 μM A23187 in culture medium for 2 h at room temperature before freezing. The YFP-tubulin transgenics were generously provided by Dr. B. Striepen [8].

### Conoid purification by differential centrifugation.

Ten replicates of conoid-enriched fraction (50 μg each) were prepared from parasite cultures harvested from a total of ~20 T175 flasks (~20 × 10^9^ parasites). For a single prep of ~2 × 10^9^, parasites were extracted twice in 1.3 ml lysis buffer (5 mM CHAPS, 1 mM Tris acetate [pH 7.5]) plus 10 mM EGTA, a mixture of protease inhibitors (1 mM TAME, 0.2 mM PMSF, 15 μM bestatin, 1 μM pepstatin A, 1 μM leupeptin, 54 μM TLCK), 2 ug/ml RNase A, and 10 u/ml DNAse I. Extracted parasite ghosts were pelleted (3 min, 12,000 × g) and washed 3 × in 1 ml lysis buffer to remove cytoplasmic proteins. After the last wash, parasite ghosts were completely resuspended in 1 ml lysis buffer and transferred to a 15-ml tube, sonicated for 5 × 2s (Heat Systems Model W-220F, Micro-tip probe, Heat Systems-Ultrasonics, Farmingdale, New York, United States), then transferred to a 1.5-ml tube and centrifuged for 3 min at 12,000 × g. The top 900 μl of supernatant (“conoid-depleted fraction”) was transferred to a fresh 1.5-ml tube. The bottom 100 μl of supernatant was discarded, and the pellet (containing >90% of the conoids) was resuspended in 1 ml lysis buffer. The suspension was then sonicated 3 × 2s as above, centrifuged and washed with lysis buffer once more without sonication to give the “conoid-enriched fraction.” Each fraction was then stored as 50μ g aliquots at −80 °C for later MudPIT analysis. Protein concentrations at each step of the preparation were measured by Bradford assay.

The two fractions were prepared for EM analysis by sedimenting at ~100,000 × g for 30 min, removing the supernatant, and fixing the pellet in 2.5% glutaraldehyde for 1 h. The fixed pellet was broken into small pieces, rinsed with 0.05 M NaHPO_4_ [pH 6.0], incubated in 1% OsO_4_ in the same buffer on ice for 30 min., rinsed, dehydrated in a graded series of ethanol then acetone, and embedded in Spurr's resin. Silver-gray sections were cut with a diamond knife, stained with uranyl acetate and lead citrate, and examined in a Phillips 400 TEM at 100kv (Royal Philips Electronics, Eindhoven, Netherlands).

### Mass spectrometry–based proteomic analysis of parasite extracts (MudPIT).

Three replicates of conoid-enriched and conoid-depleted fractions were prepared for MS/MS analysis using proteinase K digestion as described in [60]. For seven additional replicates of conoid-enriched and nine additional replicates of conoid-depleted fractions, protein solutions were brought to 400 μl in 100 mM Tris-HCl pH 8.5, precipitated with a final TCA concentration of 20% on ice overnight, washed twice with cold acetone, and dried with a SpeedVac. The protein pellets were then prepared for MS/MS analysis with endoproteinase LysC and trypsin digestion [14] as described. MudPIT was performed as described in [14,40]. MS/MS datasets were searched with a modified SEQUEST algorithm [15] against a database combining all ORFs ≥ 15aa predicted from a six-frame translation of the latest release of the T. gondii 10× genome shotgun sequences, as well as ORFs ≥ 50 aa from a six-frame translation of *Toxoplasma* EST sequences (both available at [17]), or against a database combining host (human sequences from NCBI RefSeq) with the *Toxoplasma* sequences.

The PEP_PROBE algorithm [15], a modified version of SEQUEST [61], was used to match MS/MS spectra to peptides. When applied to complex protein mixtures, the raw output from PEP_PROBE includes a large proportion of incorrect peptide assignments that must be removed by various selection criteria based on the match parameters. The PEP_PROBE outputs were parsed and filtered using DTASelect [62]. Any peptide hits had to have a minimum length of 12 amino acids. In the initial filtering, peptide assignments to spectra were retained only if they had a minimum cross-correlation score (XCorr) of 1.8 for singly-charged spectra, 2.5 for doubly-charged spectra, and 3.5 for triply charged spectra, and a normalized difference in correlation score (ΔCN) of at least 0.08. These thresholds were deliberately set low enough to be sure of retaining any bonafide spectra–peptide matches, but inevitably passed a significant number of spectra with incorrectly assigned peptides.

Further filtering was accomplished by setting up a discriminant function [16] specific for this dataset. To provide a training set, a pseudogenome was constructed, composed of 2 million random-sequence ORFs, ranging in length from 15 aa to 3000 aa (1.13 × 10^8^ total amino acids) with exactly the same length distribution and amino acid composition as the ORFs of the T. gondii genome. The truly random nature of this pseudogenome was confirmed by whole-genome BLAST against both human and T. gondii genomes. In the case of human, there were 34 and three, and for *Toxoplasma,* 121 and 20, perfectly matched 11 aa and 12 aa strings within the pseudogenome, and zero matches of longer than 12 aa in either case, as predicted for a random set of this size. The complete set of MS/MS spectra from the conoid-enriched and conoid-depleted fractions was assigned to peptides within this pseudogenome using PEP_PROBE with the same selection parameters as used for the assignments to *Toxoplasma* peptides. This yielded a set of ~14,000 peptide–spectra assignments that are known with complete certainty to be incorrect. An empirical discriminant function capable of distinguishing correct from incorrect assignments was then constructed in an iterative fashion as follows.

The incorrect peptide–spectra assignments against the random pseudogenome were combined with the observed experimental peptide assignments (an unknown mixture of correct and incorrect) against T. gondii ORFs. Initially all of the T. gondii genome peptide identifications were designated “correct.” The statistical software package STATA (StataCorp, College Station, Texas, United States) was used to generate a discriminant function for separating correct from incorrect peptide–spectra matches based on the parameters returned by PEP_PROBE [15] for each peptide identification. Of the eight parameters characterizing the peptide assignment to a spectrum, (Xcorr, ΔCN, M_H_, CalcM_H_, SpRank, SpScore, Ion Proportion, peptide length), three (M_H_, SpScore, SpRank) were found to contribute insignificantly to the discriminant function and were eliminated. Of the remaining five, Xcorr and especially ΔCN were by far the most effective. After constructing the initial discriminant function, it was used to calculate the probability that each experimental peptide identification was in fact correct. Assignments of T. gondii peptides to experimental spectra classified as having less than 20% probability of being correct in this first pass of the discriminant analysis were relabeled as “incorrect” and a new set of discriminant function coefficients was then computed. This process, which converged rapidly, was iterated until a stable distribution among “correct” and “incorrect” was obtained (typically seven to ten cycles, with very small changes beyond cycle 3). The final discriminant function correctly recognized >99% of the PEP_PROBE assignments of spectra to peptides within the pseudogenome as being incorrect.

To investigate the stability of the discriminant function, cross-validation was performed by splitting the input data (*Toxoplasma* plus pseudogenome spectra–peptide assignments) into 15 randomly selected subsets each comprising 30% of the total, and 15 separate sets of discriminant function coefficients were then iteratively calculated as above. When applied to the unused 70% of the input data, all of these functions correctly classified >99% of the incorrect peptide–spectra matches. The concordance among the classifications of individual spectra–peptide matches produced using these 15 discriminant functions averaged better than 98%. Ten independent preps of the conoid-enriched fraction and 12 preps of the conoid-depleted fraction were processed and analyzed as described above.

### Constructing a peptide–protein relational database.

Confidently identified peptides corresponding to overlapping regions from a single ORF were assembled into “contigs” using a small Fortran program written for this purpose. The complete set of 5209 nonredundant contigs was matched (see Datasets S1–S7) against the TIGR set of predicted T. gondii proteins [17] using nongapped alignment with the program FASTS34 [63] from the FASTA suite of sequence analysis software (ftp://ftp.virginia.edu/pub/fasta). The NCBI program BLASTALL (http://www.ncbi.nlm.nih.gov/BLAST/download.shtml) was used to match the contigs against the complete “nr” protein database (nonredundant protein sequence database with entries from GenPept, Swissprot, PIR, PDF, PDB, and NCBI RefSeq) and against the entire translated Plasmodium falciparum genome (all ORFs ≥ 20 aa long, http://plasmoDB.org). In all cases, the parameters used for homology search were those recommended by NCBI for finding short nearly exact matching fragments (word size two, gap opening cost nine, gap extension cost one, threshold E-value <1.0 × 10^−4^, and PAM30 scoring matrix).

### Estimating the number of missing components.

Capture–recapture analysis [45–47] of the abundance (number of spectra for each peptide in one replicate) and incidence (fraction of replicates in which a peptide was detected) data from the different replicates was carried out using the program EstimateS [45,64]. The total number of peptides or protein species present in each fraction was estimated using the Chao2 estimators with bias correction [44,65].

### Construction of fusion proteins.

1) Preparation of total parasite mRNA. RH parasites were allowed to lyse out and harvested as described above. Total mRNA of the parasites was prepared with a Qiagen RNAeasy midiprep kit (Quiagen, San Leandro, California, United States) using the protocol for mammalian cell mRNA preparation. mRNA from 5 × 10^8^ parasites (harvested from one T175 flask) was allowed to bind to one midiprep column and eluted with 150 μl elution buffer.

2) Preparation of cDNA library. 1–2 μl mRNA prepared as above was used as template in a 20 μl reaction for constructing a CDNA RACE library (Clontech SmartRace kit, Clontech, Palo Alto, California, United States).

3) Homology analysis and primer design. The complete coding sequences of the candidate genes were bootstrapped from the ORFs identified by MudPIT using overlapping fragments in the T. gondii EST database, and homology to putative gene models in *Plasmodium* or known genes in other organisms when available. Full-length coding sequences were cloned from a T. gondii cDNA library (constructed as described above) and fused with EGFP or mRFP in T. gondii expression plasmids pmin-XFP-TgGeneX. The pmin-XFP-TgGeneX are all derivatives of pmin*-*EGFP-TgCentrin1 [7] in which expression of the protein is controlled by the *T. gondii* DHFR-TS promoter [59]. To create pmin-mRFP-TgCentrin1, the mRFP coding sequence was amplified from vector pRSET-mRFP1 [66] by PCR with primers:

5′-TCTGgctagcAAAATGGCCTCCTCCGAGGACGTCATCAA-3′

5′-ATCGagatctGGCGCCGGTGGAGTGGCGGCCCTCG-3′

digested with NheI and BglII and ligated in place of EGFP.

For construction of pmin*-*XFP-GeneX, the GeneX coding sequences were amplified from the cDNA library by PCR with the primers shown in Table 3.

**Table 3:**
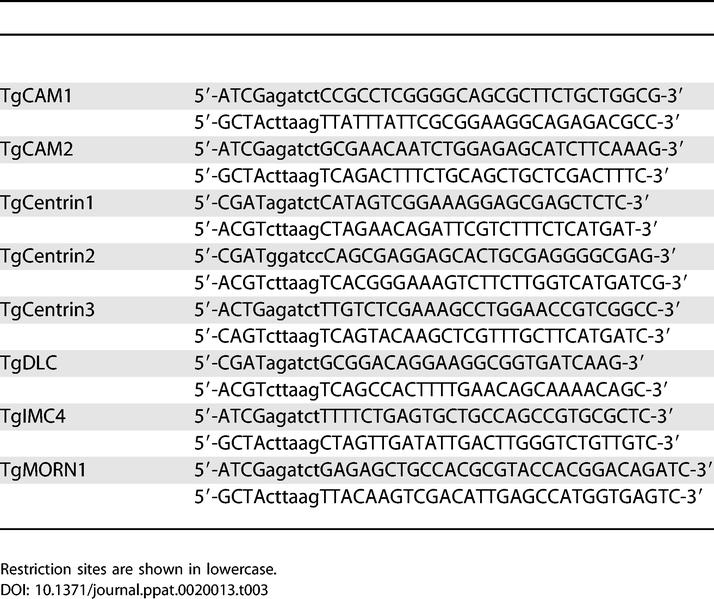
Primers Used for PCR Amplification of the cDNAs Corresponding to the Proteins Discussed in the Text

### Stable transgenics and clones.

There is no eukaryotic selectable marker on the pmin-XFP-GeneX plasmids. Stable transgenic parasite lines were generated by cotransfection with plasmid pC3, which carries a mutated DHFR-TS gene, conferring pyrimethamine resistance to the transfected parasites [67]). 10^7^ RH parasites were transfected by electroporation with 45 μg of pmin*-*GFP-TgGeneX and 5 μg of PC3 and allowed to infect a monolayer of host cells. To produce stable transgenics, pyrimethamine was added 15–20 h later to a final concentration of 1 μM, and drug-resistant clones expressing XFP-TgGeneX were isolated by limiting dilution after several rounds of selection. Clones with intermediate levels of fluorescence were chosen for further characterization.

### Light microscopy.

Fixed cells were prepared and observed as previously described [27,68]. For live-cell imaging and time-lapse microscopy, parasites were inoculated into a subconfluent layer of human foreskin fibroblast cells grown in a 35-mm plastic dish with #1.5 glass coverslip bottom (MatTek #P35G-1.5–10-C, MatTek, Ashland, Massachussetts, United States). Immediately before use, the medium was replaced with CO2-independent medium or DMEM plus 25 mM HEPES (Gibco #18045–088 or 12320–032, Gibco, San Diego, California, United States). Temperature and humidity were controlled by means of a box completely surrounding the microscope as described [69], which also eliminated the focus drift due to temperature fluctuations that otherwise bedevil long time-lapse experiments. 3D image stacks were collected at z-increments of 0.3 μm on an Applied Precision DeltaVision workstation (Applied Precision, Issaquah, Washington, United States) based on an Olympus IX-70 inverted microscope (Olympus, Tokyo, Japan), using a 60× NA 1.2 water immersion or 60× NA 1.4 oil-immersion lens. Brightly fluorescent 0.2 μm beads placed in the dish were used to adjust the correction collar (water-immersion lens) or refractive index of the immersion oil (oil-immersion lens) to minimize spherical aberration. Deconvolved images were computed using the point-spread functions and software supplied by the manufacturer. In the final displayed images, the relative brightness of different fluorophores has been altered when necessary to match the gamut of a color printer.

### Immunogold labeling.

Transgenic parasites (from one T25 flask, 2 × 10^7^ to 5 × 10^7^) expressing EGFP-TgGeneX were resuspended in 30-μl permeabilization buffer (either 10 mM deoxycholate in dH2O, or 0.5% TritonX-100 in 0.2 × PBS with 0.3-unit DNaseI) (Stratagene, La Jolla, California, United States) and rotated at 20 °C for 1 h (EGFP-TgDLC immunoEM) or 5min (all others). 3-μl-extracted parasite suspension was allowed to settle on a carbon-film–coated nickel grid for 10 min in a humid chamber. Grids were washed 3× with water (1min, 5min, 15 min, respectively). Parasites were then fixed 10 min with 3.7% formaldehyde in PBS, and washed 3 × 5 min with PBS, followed by 10-min blocking in 5% BSA + 0.1% fish gelatin. Free aldehyde groups were blocked by incubation with 50 mM glycine in PBS for 10–30 min (pH 7.5) followed by 10–20 min incubation with 0.1% NaBH_4_ in PBS. Grids were washed twice with PBS, blocked again with 5% BSA + 0.1% fish gelatin in PBS for 30 min, washed 2 × 5 min with incubation buffer (0.8%BSA, 0.1% fish gelatin in PBS plus 15 mM NaN3), incubated for ~3 h with primary antibody (rabbit anti-GFP polyclonal, a kind gift from Dr. Graham Warren, Yale University, New Haven, Connecticut, United States), diluted 1:8000 in incubation buffer; washed 6 × 5 min in incubation buffer, inverted on 10-μl drops of secondary antibody (anti-rabbit IgG conjugated with 1.4 nm gold, Nanoprobes, Yaphank, New York, United States), diluted 1:160 in incubation buffer; incubated 18–36 h at 4 °C, then washed with incubation buffer as follows: 3 × 1 min, 2 × 10 min, 4 × 5 min, and then 5 × 1 min with PBS. The samples were post-fixed 5 min with 1% glutaraldehyde in PBS and washed in distilled water 3 × 5 min. Silver enhancement was carried out using the HQ silver enhancement kit (Nanoprobes) by floating grids on mixtures of the initiator, activator, and modulator for 2–3 min in a light-tight chamber, then washing briefly with dH2O once followed by 2 × 5 min wash. Grids were negatively stained using 2% phosphotungstic acid (pH 7.0) mixed with ~ 0.5 mg/ml cytochrome C.

## Supporting Information

Dataset S1 List of Proteins Found in the Conoid-Enriched Fraction Only (36 KB TXT)

Dataset S2 List of Proteins Found in the Conoid-Enriched Fraction Only but Omitting Proteins Identified as Contaminants Derived from Other Subcellular Organelles (16 KB TXT)

Dataset S3 List of Proteins Found in the Conoid-Depleted Fraction Only (23 KB TXT)

Dataset S4 List of Proteins Found in the Conoid-Depleted Fraction Only but Omitting Proteins Identified as Contaminants Derived from Other Subcellular Organelles (13 KB TXT)

Dataset S5 List of Proteins Found in Both the Conoid-Enriched and Conoid-Depleted (18 KB TXT)

Dataset S6 List of Proteins Found in Both the Conoid-Enriched and Conoid-Depleted Fractions but Omitting Proteins Identified as Contaminants Derived from Other Subcellular Organelles (10 KB TXT)

Dataset S7 Peptide Contigs Identified by MudPIT (493 KB TXT)

Protocol S1 List of Proteins and Peptides Identified in Each Fraction (28 KB DOC)

Video S1 EGFP-DLC in T. gondii after Treatment with Calcium Ionophore A monolayer culture of human fibroblast cells was infected with a transgenic line of the obligate intracellular parasite T. gondii. The parasites express EGFP-TgDLC, which was imaged by epifluorescence microscopy. Bright fluorescence is seen in the middle of the cell at the centriole/spindle pole, in an apical bright spot at the conoid, and as diffuse fluorescence with a brighter cap at the apical end of the cell. In the video, several parasitophorous vacuoles are seen, each containing two parasites. The nonfluorescent host fibroblasts are not visible. The video sequence commences with addition of Ca^2+^ ionophore A23187 to the culture medium. After a short delay, the parasites begin egressing from the host cells by vigorous gliding movements, coupled with protrusion of the conoid. An image of one cell, indicated by the orange arrow, has been digitally cut out, rotated into constant orientation, and shown as an enlargement within the box at the upper right. Sudden protrusion of the conoid is particularly clear in this parasite, just after it begins to glide. Note that in this parasite and several others, a waist-like constriction is observed to pass down the cell as it squeezes through the host cell membrane. Some parasites display this twice, once as they exit the host cell and then again as they reinvade a second host.
The images were collected at 2-s intervals, at room temperature. The total elapsed time for the video sequence is 288 seconds.
(2.8 MB MOV)

Video S2 Cell Division in T. gondii
A monolayer culture of human fibroblast cells was infected with a transgenic line of the obligate intracellular parasite T. gondii. The parasites express EGFP-IMC1 (green), a marker for the mother and daughter IMC, and mRFP-TgMORN1 (red). DIC and epifluorescence images are shown in the video. Several parasitophorous vacuoles are seen, containing two or four parasites each. The nonfluorescent host fibroblasts are seen in the DIC image only. 3D stacks of images were collected at 0.6 μ increments in z, every 10 min. The DIC image is a single plane from the middle of the stack. The epifluorescence image is a projection of the deconvolved 3D stack. The total elapsed time for the video sequence is 16 h 40 min.
(4.4 MB MOV)

## Abbreviations

DLC: dynein light chain
IMC: inner membrane complex
MORN: membrane occupation and recognition nexus
MT: microtubules
MudPIT: Multidimensional Protein Identification Technology
ORF: open reading frame
