# Supplementary material for: Cytoskeletal Components of an Invasion Machine—The Apical Complex of Toxoplasma gondii
Source: PLoS Pathog. 2006 Feb 24;2(2):e13. doi: 10.1371/journal.ppat.0020013 (PMC1383488; doi:10.1371/journal.ppat.0020013)
Supplement: Protocol S1 — (28 KB DOC) [file ppat.0020013.sd008.doc]

**Protocol S1.**

**List of proteins and peptides identified in each fraction**

Seven tab-delimited text files:

con_enr_only.tab 288 rows, 6 columns

con_enr_nocontam.tab 180 rows, 6 columns

con_dep_only.tab 354 rows, 6 columns

con_dep_nocontam.tab 238 rows, 6 columns

both_.tab 524 rows, 6 columns

both_nocontam.tab 266 rows, 6 columns

all_contigs.tab 5023 rows, 8 columns

The first 3 pairs of files list the proteins found in the conoid-enriched fraction only ("con_enr_only..."), conoid-depleted fraction only ("con_dep_only.."), or both ("both..."). One file of each pair lists all proteins found in the fraction. The second file omits proteins identified as contaminants derived from other sub-cellular organelles ("..._nocontam.tab"). Each file begins with a row of column labels. The columns are:

TIGR ID *T. gondii* Draft 3 Annotation gene/predicted protein ID; early draft kindly provided by Aaron Mackey and David Roos, Genomics Institute, Univ. of Pennsylvania and Ian Paulsen, TIGR. (http://toxodb.org/ToxoDB.shtml)

length number of amino acids in predicted protein

%coverage percentage of amino acids in MudPIT-identified peptides

# of contigs number of non-overlapping peptide fragments identified

TwinScan ID gene annotation by TwinScan (http://toxodb.org/ToxoDB.shtml)

Description function or homologue in other organisms, if known

The seventh file lists all peptide "contigs" identified by MudPIT. The columns are:

TIGR ID *T. gondii* Draft 3 Annotation gene/predicted protein ID; early draft kindly provided by Aaron Mackey and David Roos, Genomics Institute, Univ. of Pennsylvania and Ian Paulsen, TIGR. (http://toxodb.org/ToxoDB.shtml)

TIGR_start position of first aa in predicted protein

Twinscan ID Twinscan predicted protein ID (http://toxodb.org/ToxoDB.shtml)

Twinscan_start position of first aa in predicted protein

Locus ID genomic or EST identifier

Locus_start position of first aa in locus

Locus end position of last aa in locus

contig length number of amino acids in contig

con_enr_spect number of spectra in conoid-enriched fraction

con_enr_rep number of replicates of conoid-enriched fraction in which this contig appeared

con_dep_spect number of spectra in conoid-depleted fraction

con_dep_rep number of replicates of conoid-depleted fraction in which this contig appeared

# of peptides number of peptides contributing to this contig

sequence aa sequence of contig
